# Tracheal leiomyoma imitating refractory asthma: A case report

**DOI:** 10.1016/j.ijscr.2023.108006

**Published:** 2023-03-20

**Authors:** Ershadi Reza, Amini Hesam, Soltanmohammadi Sara, Rafieian Shahab

**Affiliations:** a Department of Thoracic Surgery, Imam Khomeini Hospital Complex, Tehran University of Medical Sciences, Tehran, Iran; b Department of Thoracic Surgery, Imam Khomeini Hospital Complex, Tehran University of Medical Sciences, Tehran, Iran; c Department of Pulmonology, Imam Khomeini Hospital Complex, Tehran University of Medical Sciences, Tehran, Iran; d Department of Thoracic Surgery, Imam Khomeini Hospital Complex, Tehran University of Medical Sciences, Tehran, Iran

**Keywords:** Tracheal leiomyoma, Surgical resection, Refractory asthma, Airway obstruction, Fiberoptic bronchoscopy

## Abstract

**Introduction and importance:**

Tracheal leiomyoma is an extremely rare benign tumor. It mostly presents in the third decade of life and mostly affects men. Herein, we describe a patient with tracheal leiomyoma which was treated as asthma for 2 years before definite diagnosis.

**Case presentation:**

A 41-year-old female with a history of asthma was referred due to dyspnea and refractory cough. On bronchoscopic examination, a tumoral lesion was found in the distal trachea with near total obstruction and histopathologic examination of the bronchoscopic biopsy was inconclusive. The tumor was surgically resected. On the follow-up bronchoscopic examination, the trachea was normal and symptoms were relieved. Histopathologic results were compatible with Leiomyoma.

**Clinical discussion:**

Airway leiomyoma is commonly misdiagnosed as asthma or bronchitis long before a definitive diagnosis. Fiberoptic bronchoscopy is the modality of choice for direct visualization of intraluminal lesions and tissue sampling. Surgical resection is the gold standard approach. The best surgical approach is not clearly determined to date and both endoscopic procedures and surgical resection have been utilized for treatment in case reports.

**Conclusion:**

Usually there is a long interval between onset of clinical symptoms and a definite diagnosis. In the case of refractory signs and symptoms to medical treatment, alternative diagnosis should always be considered.

## Highlights

• Tracheal leiomyoma is an extremely rare benign tumor.
• A 41-year-old female with a history of asthma was referred due to dyspnea and refractory cough.
• On bronchoscopic examination, a tumoral lesion was found in the distal trachea with near total obstruction.
• Surgical resection is the gold standard approach.

## 1 Introduction

Airway leiomyoma is an extremely rare benign tumor of bronchial tree which was first described in 1955 [1]. It accounts for <2 % of benign tumors of the lower airway [2] and tracheal leiomyoma is about 1 % of all tracheal tumors [3]. It can occur at any age but mostly presents at third decade of life. Lung parenchymal leiomyoma is more prevalent in women while airway leiomyoma mostly affects men [2]. Clinical findings depend on the size and location of the tumor, from completely asymptomatic to life threatening airway obstruction. We present a case of tracheal leiomyoma with obstructive features. This work has been reported in line with the SCARE criteria [4].

## 2 Presentation of case

**Fig. 1:**
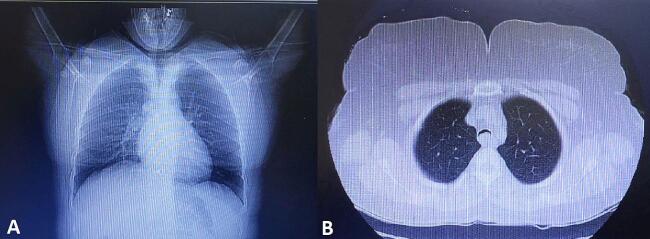
CT-scan. Scout view showing tracheal narrowing above the carina (A). Axial view showing intraluminal tracheal mass lesion (B).

**Fig. 2:**
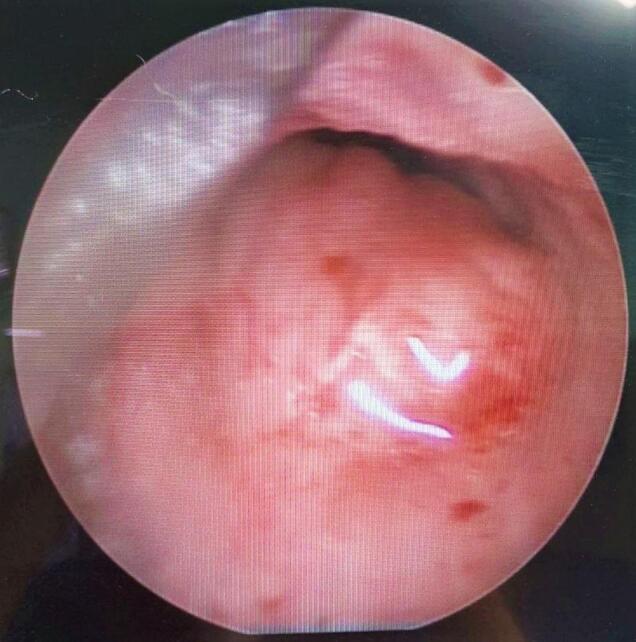
Bronchoscopic view of the endotracheal tumor.

**Fig. 3:**
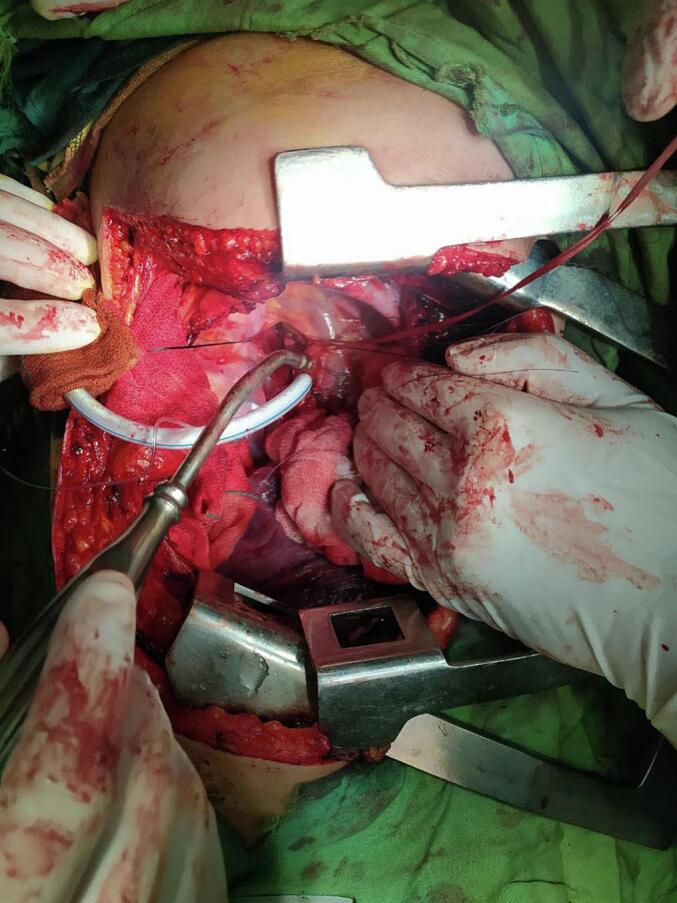
Thoracotomy.

**Fig. 4:**
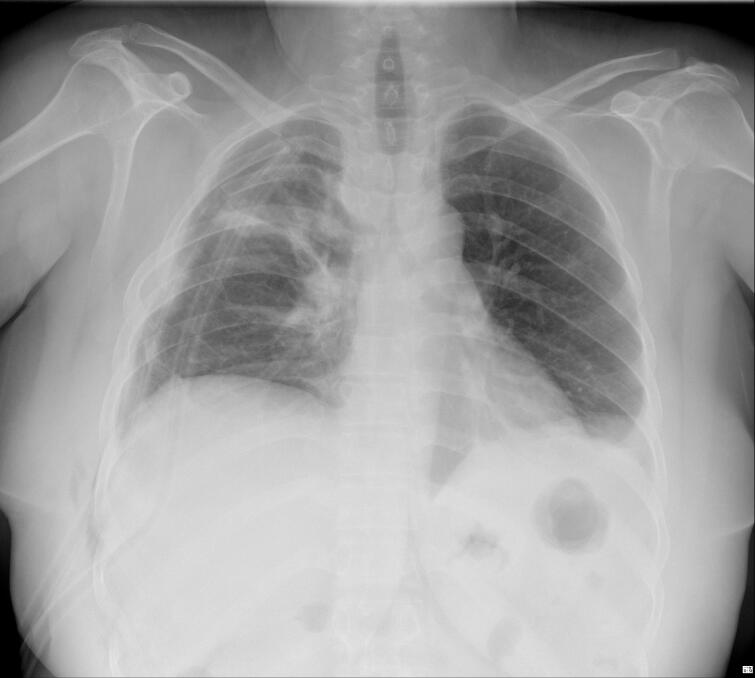
Post-operation CXR.

**Fig. 5:**
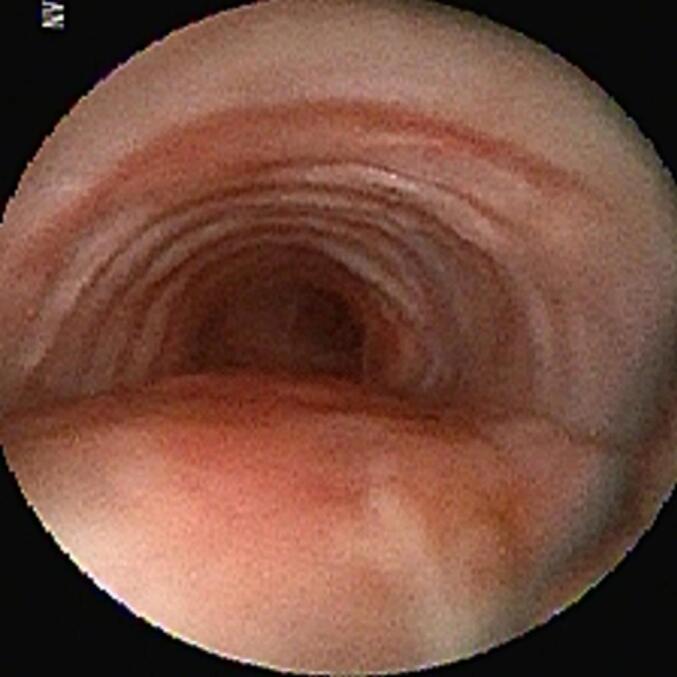
Follow up bronchoscopy revealing normal trachea.

**Fig. 6:**
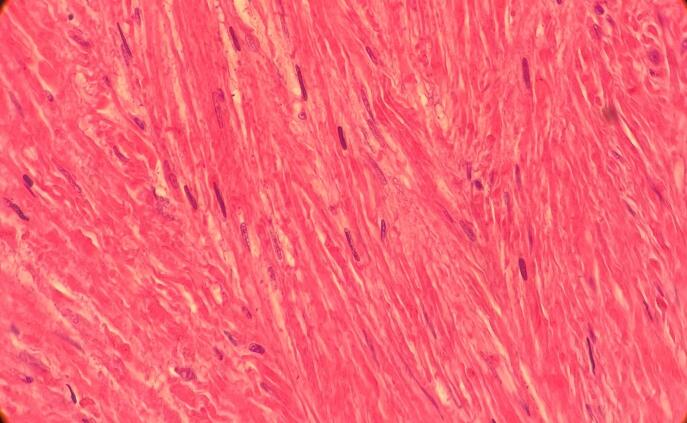
Photomicrograph showing spindle cells with cigar shaped nuclei (H&E).

A 41-year-old woman was referred with dyspnea and refractory cough from another general hospital. The thoracic surgeon had evaluated the patient and found a tumoral lesion close to the carina in fiber optic bronchoscopy. Histopathologic evaluation of the tissue biopsy was inconclusive. Her medical history included asthma diagnosed 2 years earlier based on spirometry: FEV1/FVC: 64, FEV1: (1.78) 65 %, FVC: (2.78) 83 %. She was receiving Budesonide/Formoterol 320–9 μg MDI 2 puffs twice daily for controlling asthma. Despite good adherence to treatment, there was no significant improvement in symptoms and her condition has progressively gotten worse during the past 2 months. She was not a smoker and denied secondhand smoking. Her allergic history and family history was also unremarkable. Her initial vital signs were BP 115/80 Hg, pulse rate 90 bpm, respiratory rate 22 pm, temperature 37.1. On admission in thoracic surgery ward, she had inspiratory and expiratory stridor in the physical examination. The patient had a chest computed tomography scan which was carried out in the first hospital, showing a tracheal mass close to the carina (Fig. 1). Possible complications of the operation were explained to the patient and her family member and the possibility of needing a thoracotomy for resection of the mass was pointed out to her. On fiber optic bronchoscopy, a smooth polypoid tumor of 18 mm in diameter arising from the membranous part of the trachea was discovered approximately 10 cm distal to the vocal cords and 1 cm proximal to the carina (Fig. 2). The trachea was near totally occluded by the tumor. The lesion was firm and was not resectable via wire snare electro cautery. Due to the patient's symptoms and occlusion of the trachea, urgent surgery was planned. The patient got intubated with a single lumen tracheal tube by fiberoptic bronchoscopy guidance. The patient was placed in the left lateral decubitus position. A right posterolateral incision through the 4th intercostal space was made. The carina explored and exposed. The tumor was palpable 1 cm proximal to the carina. A longitudinal incision on the membranous part of the trachea was made. A single lumen tracheal tube inserted in the distal part of the trachea (Fig. 3). Because of long segment involvement of trachea by the tumor, and for prevention of tracheal stenosis, only the membranous portion of trachea was resected. An end-to-end anastomosis was done, and the endotracheal tube was removed. The incision got closed after insertion of 28 French chest tube. Post-operation CXR is illustrated in (Fig. 4). The postoperative bronchoscopy was satisfying. There was no evidence of uterine leiomyoma in pelvic ultrasonographic study. The patient was discharged one week after surgery with normal bronchoscopy and no signs and symptoms of tracheal stenosis and antitussive drug was prescribed. After three months, the patient visited and a fiberoptic bronchoscopy was done which was normal (Fig. 5) and dyspnea and cough were completely relieved and the patient was satisfied with the results. Histopathologic evaluation of the tumor revealed spindle cells with cigar shaped nuclei with the greatest dimension of 1.4 cm and mitosis 0–1/10HPF (Fig. 6). Immunohistochemistry assessment showed: SOX10: Negative, Desmin: Positive, SMA: Positive-CKit: Negative, DOG1: Negative, Ki67: 1 %, which was compatible with leiomyoma.

## 3 Discussion

Primary leiomyoma of airway is a scarce disease and has been described only as case reports. Distal part of tracheobronchial tree is affected more than the proximal part and the lower third of the trachea is involved the most and it commonly originates from posterior membranous wall [5], [6]. The majority of leiomyomas arising from lung parenchyma are asymptomatic and they are found as an incidental finding in radiologic studies [7]. Bronchial leiomyoma is frequently symptomatic due to partial or total obstruction of affected segment and produce symptoms such as hemoptysis, cough, dyspnea, sputum production and fever as the result of post-obstructive pneumonia, atelectasis, wheezing, etc. [8] Due to shared clinical features, airway leiomyoma is commonly misdiagnosed as asthma or bronchitis long before definitive diagnosis [9].

Leiomyoma doesn't have any pathognomonic radiologic findings. Tracheal masses are hardly ever visible on plain chest radiographs [10]. Computed tomography (CT) findings of leiomyoma are nonspecific: an intraluminal smooth mass with soft tissue density (25–46 Hounsfield units on non-contrast CT and 46–85 Hounsfield units on contrast CT) [7], [11], atelectasis, unilateral emphysema or hyperlucency because of air trapping distal to obstructed bronchus [12]. A few cases of leiomyoma calcification have also been reported [2].

Fiberoptic bronchoscopy is the modality of choice for direct visualization of intraluminal lesions and tissue sampling. In the case of complete obstruction, airway patency distal to the stenosis cannot be assessed by bronchoscopy. Hence, 3-D reconstruction of high-resolution CT can help further investigations [2], [7]. endoscopic samples cannot always differentiate between leiomyoma and leiomyosarcoma. Therefore, wider surgical resection is sometimes necessary [13]. In pathologic examination, spindle-shaped cells are evident, and Immunohistochemistry can aid definite histologic diagnosis [14]. Malignancy possibility should be strongly regarded in the case of increased mitotic activity (>3/10 high-power fields), cytologic atypia, and necrosis [15].

Medical treatment is ineffective and surgical resection is the gold standard approach. Prognosis is excellent with complete removal of the tumor. The best surgical approach is not clearly determined to date. Tracheal sleeve resection, carinal resection, endoscopic resection, Nd-YAG laser ablation, electrocoagulation, cryotherapy, have been employed in case reports. Endoscopic resection and ablation can result in complications such as tracheal perforation, positive surgical margin, and lost removed tumor [16].

## 4 Conclusion

Tracheal leiomyoma is a rare benign airway tumor. Due to its rarity, there is a long interval between onset of clinical symptoms and a definite diagnosis. In the case of refractory signs and symptoms to medical treatment, alternative diagnosis should always be considered, and further investigation should be attempted.

## Consent

Written informed consent was obtained from the patient for publication of this case report and accompanying images. A copy of the written consent is available for review by the Editor-in-Chief of this journal on request.

## Ethical approval

Ethical approval was waived by the authors institution.

## Funding

N/A.

## Guarantor

Amini Hesam.

## CRediT authorship contribution statement

Amini H: Conceptualization, Writing - Original draft, Investigation, Resources.
Soltanmohammadi S: Methodology, Validation, Supervision, Writing - Review and editing.
Rafieian Sh and Ershadi R: Writing - Original draft, Resources.

## Declaration of competing interest

N/A.

## References

[1] Dorenbusch A.A. (1955). Leiomyoma of the trachea: a case report. AMA Arch. Otolaryngol..

[2] White S.H., Ibrahim N.B., Forrester-Wood C.P., Jeyasingham K. (1985). Leiomyomas of the lower respiratory tract. Thorax.

[3] Sugiyama M., Yoshino I., Shoji F., Hamatake M., Yohena T., Osoegawa A., Maehara Y. (2009). Endotracheal surgery for leiomyoma of the trachea. Ann. Thorac. Cardiovasc. Surg..

[4] Agha R.A., Franchi T., Sohrabi C., Mathew G., for the SCARE Group (2020). The SCARE 2020 guideline: updating consensus Surgical Case Report (SCARE) guidelines. International Journal of Surgery.

[5] Sanders J.S., Cornes V.M. (1961). Leiomyoma of the trachea. Report of a case, with a note on the diagnosis of partial tracheal obstruction. N. Engl. J. Med..

[6] Foroughi E. (1962). Leiomyoma of the trachea. Dis. Chest.

[7] Taylor T.L., Miller D.R. (1969). Leiomyoma of the bronchus. J. Thorac. Cardiovasc. Surg..

[8] Cárdenas-García J., Lee-Chang A., Chung V., Shim C., Factor S., Tibb A. (2014). Bronchial leiomyoma, a case report and review of literature. Respir. Med. Case Rep..

[9] Harrison O.J., Jackson M., Shaw E., Alzetani A. (2020). Tracheal leiomyoma mimicking asthma for over 20 years. J. Surg. Case Rep..

[10] Douzinas M., Sheppard M.N., Lennox S.C. (1989). Leiomyoma of the trachea—an unusual tumour. Thorac. Cardiovasc. Surg..

[11] Ko J.M., Jung J.I., Park S.H., Lee K.Y., Chung M.H., Ahn M.I. (2006). Benign tumors of the tracheobronchial tree: CT-pathologic correlation. AJR Am. J. Roentgenol..

[12] Sharifi N., Massoum S.H., Shahri M.K., Rezaei A., Ashari A.A., Attar A.S. (2010). Endobronchial leiomyoma; report of a case successfully treated by bronchoscopic resection. J. Res. Med. Sci..

[13] Yellin A., Rosenman Y., Lieberman Y. (1984). Review of smooth muscle tumours of the lower respiratory tract. Br. J. Dis. Chest.

[14] Miller D.R. (1969). Benign tumors of lung and tracheobronchial tree. Ann. Thorac. Surg..

[15] Kim Y.K., Kim H., Lee K.S., Han J., Yi C.A., Kim J., Chung M.J. (2007). Airway leiomyoma: imaging findings and histopathologic comparisons in 13 patients. Am. J. Roentgenol..

[16] Gupta V., Vishwakarma R., Patel K., Desai H. (2013). Tracheal leiomyoma: a clinical dilemma. J. Laryngol. Voice.

